# Multitarget Anti‐*Candida* Activity of Thai Plant Extracts and Essential Oils: Inhibiting Biofilm Formation, Denture Adhesion, and Germ Tube Formation

**DOI:** 10.1155/sci5/1766872

**Published:** 2025-12-10

**Authors:** Premnapa Sisopa, Supaporn Lamlertthon, Ruchadaporn Kaomongkolgit, Pratthana Chomchalao, Waree Tiyaboonchai

**Affiliations:** ^1^ Department of Pharmaceutical Technology, Faculty of Pharmaceutical Sciences, Naresuan University, Phitsanulok, 65000, Thailand, nu.ac.th; ^2^ Department of Health and Cosmetic Product Development, Faculty of Food and Agricultural Technology, Pibulsongkram Rajabhat University, Phitsanulok, 65000, Thailand, psru.ac.th; ^3^ Department of Microbiology and Parasitology, Faculty of Medical Science, Naresuan University, Phitsanulok, 65000, Thailand, nu.ac.th; ^4^ The Center of Excellence in Medical Biotechnology, Naresuan University, Phitsanulok, 65000, Thailand, nu.ac.th; ^5^ Department of Oral Diagnosis, Faculty of Dentistry, Naresuan University, Phitsanulok, 65000, Thailand, nu.ac.th; ^6^ College of Medicine and Public Health, Ubon Ratchathani University, Ubon Ratchathani, 34190, Thailand, ubu.ac.th; ^7^ Center of Excellence for Innovation in Chemistry (PERCH-CIC), Naresuan University, Phitsanulok, 65000, Thailand, nu.ac.th

**Keywords:** adhesion, biofilm, *Candida albicans*, essential oil, germ tube, plant extract

## Abstract

This study aimed to evaluate the efficacy of Thai plant extracts (PEs) and essential oils (EOs) against reference and clinical isolate strains of *Candida albicans,* focusing on their ability to inhibit biofilm formation, cell adhesion to denture acrylic, and germ tube formation. The minimum biofilm inhibition concentration (MBIC) and the minimum biofilm eradication concentration (MBEC) were determined. The impact on adhesion to denture acrylic was determined by XTT assay, and germ tube inhibition was evaluated using the counting chamber. The results revealed that cinnamon bark oil exhibited the lowest MBIC_90_ and MBEC_90_ values (0.156 mg/mL and 0.313 mg/mL, respectively) against both *C. albicans* strains, followed by lemongrass oil, clove bud oil, *Alpinia galanga* extract, and *Piper betle* extract. A similar inhibitory trend was observed for cell adhesion to denture acrylic and germ tube formation. In particular, *A. galanga* extract (2.50 mg/mL) significantly reduced *C. albicans* adhesion to denture acrylic by over 80%. Additionally, cinnamon bark oil, lemongrass oil, and *A. galanga* extract could inhibit the germination of *C. albicans* at 0.5×MIC. In conclusion, this study indicates that all tested agents possessed anti–*C. albicans* biofilm activity through decreasing adhesion and yeast–hyphae transition of *C. albicans* cells. Therefore, these EO and PE could serve as alternative antifungals for treating oral candidiasis.

## 1. Introduction

Denture stomatitis (DS) is oral candidiasis caused by an infection and *Candida* overgrowth, mainly *Candida albicans*, in denture wearers. This pathogen forms biofilms on dentures and oral mucosa, leading to chronic inflammation and redness of the supporting membrane [1, 2]. This persistent biofilm is critical for the frequent and rapid recurrence of DS following treatment cessation [3]. Though often asymptomatic, DS can cause discomfort, including a burning sensation, soreness, and difficulty eating, significantly impacting a patient’s quality of life. Effective management of DS typically involves proper oral hygiene and antimicrobials such as chlorhexidine and sodium hypochlorite, alongside removing dentures at night [4]. For recurrent DS, antifungal drugs (e.g., fluconazole, nystatin, and amphotericin B) are often prescribed [5, 6]. Despite their use, these chemical agents carry limitations including staining, altered taste, skin irritation, and the critical issue of developing drug‐resistant strains [7–9]. This highlights a need for novel, safe antifungal agents with strong antibiofilm potential for effective DS management.

Several studies highlighted the potential of natural products in combating *Candida* biofilm, such as lemongrass oil, clove bud oil, cinnamon bark oil, betel *(Piper betle)* extract, and pomegranate extract [10–12]. Clove oil has been reported to inhibit growth, transition, and biofilm formation by downregulating *HWP1*, *AlS3*, and *SAP3* gene expression, which is responsible for the resistance of *C. albicans* [13]. In our previous studies, three essential oils (EOs) (lemongrass, clove bud, and cinnamon bark) and two plant extracts (PEs) (*P. betle* extract and *Alpinia galanga* extract) have been investigated for their antimicrobial activity against *C. albicans* ATCC1023 and clinical isolates. These EO and PE exhibited potent antifungal activity against *C. albicans*, with minimum inhibitory concentration (MIC) ranging from 0.078 to 1.250 mg/mL for the reference strain and 0.156 to 2.500 mg/mL for clinical isolates [14]. Moreover, the major bioactive compounds identified from these EO and PE also exhibited strong inhibition against *Candida* species.

Based on their promising anticandidal properties, this study aimed to evaluate the ability of EO and PE to prevent *C. albicans* biofilm formation, eradicate established biofilms, inhibit yeast cell adhesion to denture acrylic surfaces, and suppress germ tube formation (GTF).

## 2. Materials and Methods

### 2.1. Strains and Culture Conditions

The *C. albicans* strains used were ATCC10231 (reference) and R01 (clinical isolate). *C. albicans* ATCC10231 was obtained from the Faculty of Medical Sciences, Naresuan University. The clinical isolate (*C. albicans* R01) was acquired from the Faculty of Dentistry at Naresuan University, which was collected from pseudomembranous candidiasis patients attending the Oral Diagnosis and Oral Medicine Clinic of the Dental Hospital at Naresuan University, Thailand. These two strains were stored as frozen stock cultures in the culture medium (yeast extract–peptone–dextrose [YPD]) supplemented with 10% (v/v) of glycerol at −80°C. The stock culture was subcultured onto Sabouraud dextrose agar (SDA; HiMedia, India) plates and incubated at 37°C for 24 h before performing antifungal activity tests.

### 2.2. Preparation of PEs and EOs

*P. betle* leaves and *A. galanga* were collected locally in Phitsanulok, Thailand. The dried plant was milled and underwent a 3‐day maceration extraction with 95% ethanol at ambient temperature. Each extract was filtered and concentrated using a rotary evaporator (Buchi, Switzerland) and kept at −20°C. Before antifungal testing, each PE was reconstituted to 10 mg/mL using a mixture of 4% dimethyl sulfoxide (DMSO) and 4% Tween 80.

EOs from lemongrass, clove bud, and cinnamon bark were purchased from Thai China Flavours & Fragrances Industry Co., Thailand. Each EO was diluted to 20 mg/mL with 4% Tween 80 before antifungal activity testing.

### 2.3. Inhibitory Effect on *Candida* Biofilm Formation

Each *C. albicans* strain suspension was prepared from an overnight culture in 20 mL of Sabouraud dextrose broth (SDB; HiMedia, India) at 37°C for 24 h. Cells were collected by centrifugation and washed twice with sterile phosphate‐buffered saline (PBS; Sigma‐Aldrich, USA) and resuspended in RPMI‐1640 medium (Gibco™, Austria) to approximately 1 × 10^6^ cells/mL [15].

The inhibitory effects of EO and PE on *C. albicans* biofilm formation were determined by the modified microdilution method [16]. Initially, 100 μL of the sample was twofold serially diluted with RPMI‐1640 in a 96‐well plate, yielding a concentration range of 0.02–5.00 mg/mL. An equal volume of *C. albicans* suspension was individually added and mixed with the sample (test). The plate was incubated at 37°C for 48 h to allow biofilm formation. Then, the medium was aspirated, and nonadherent cells were removed by washing thrice with sterile PBS. The biofilm quantity was determined by sodium 3′‐[1‐[(phenylamino)‐carbonyl]‐3,4‐tetrazolium]‐bis (4‐methoxy‐6‐nitro) benzenesulfonic acid reduction assay (XTT; Abcam, USA) as described below. Chlorhexidine ranging from 1.0 to 250 μg/mL was used as a positive control. The selection of CHX concentrations was based on the established MIC/MFC value (31 μg/mL) determined against a clinical *C. albicans* strain in our previous study [14]. *C. albicans* in RPMI‐1640 was designated as the control. The minimum biofilm inhibition concentration (MBIC_90_) was defined as the lowest concentration that inhibited 90% of fungal biofilm growth. All tests were performed in triplicate and repeated on three separate occasions. The percentage of biofilm inhibition was calculated using the following formula:

(1) % Inhibition=1−OD490 nm testOD490 nm control×100%.

### 2.4. Eradication Activity Against Established Mature *Candida* Biofilm

An eradication activity of EO and PE against established *C. albicans* biofilm was carried out as previously described [16]. Briefly, 200 μL of *Candida* suspension (∼1 × 10^6^ cells/mL) was inoculated into a 96‐well plate and incubated for 48 h at 37°C for mature biofilm formation. The medium was then aspirated, and each well was carefully washed three times with PBS to remove nonadherent cells. The established fungal biofilm was then exposed to 200 μL of plant sample ranging from 0.02 to 5.0 mg/mL and incubated for 48 h at 37°C. After treatment, the treated and nontreated fungal biofilms were washed three times with PBS. The viability of EO‐ or PE‐treated biofilm was determined by XTT reduction. CHX was used as the positive control. Biofilm in a sample‐free medium served as the control. The minimum biofilm eradication concentration (MBEC_90_) was defined as the lowest concentration that eliminated the mature biofilm by 90%. Each test was performed in triplicate, and all experiments were repeated over three different periods. The percentage of biofilm eradication was calculated using the following formula:

(2) % Eradication=1−OD490 nm tested biofilmOD490 nm control×100%.

### 2.5. Effect on Yeast Cell Adhesion

Acrylic strips were prepared according to the previous study [17]. Self‐polymerizing acrylic powder and monomer liquid were mixed following the manufacturer’s instructions (Takilon, Rodent, s.r.l., Milano, Italy). The mixture was sandwiched between two glass slides (2.5 cm × 7.5 cm) and fastened at both ends by two clips. The acrylic was then polymerized at 50°C for 5 min in a hydroflask. Then, the obtained transparent acrylic sheet was removed and sliced into strips (5 mm × 5 mm square and 0.4 mm thick). To remove excess monomer, these strips were soaked in distilled water for 1 week, dipped in 70% alcohol for 1 min, and then rinsed three times in distilled water. Finally, they were sealed and sterilized before use in the adhesion assay.

The effect of EO and PE on yeast cell adhesion to the acrylic strip was performed as previously described [17]. Acrylic strips were individually placed in a 48‐well plate, with each well primarily containing 0.2 mL of EO or PE at specified concentrations (MIC, 2×MIC, and 4×MIC). Then, 0.2 mL of *C. albicans* suspension (10^8^ cells/mL) prepared in RPMI‐1640 medium was added to each well and incubated for 3 h at 37°C with gentle agitation at 120 rpm/min. After incubation, acrylic strips were washed three times with PBS to remove nonadherent cells and transferred to new 48‐well plates. The fungal cell viability was determined using an XTT reduction assay. CHX served as the positive control, whereas *C. albicans* in the EO or PE‐extract‐free medium served as the control. All tests were performed in triplicate, and the experiments were repeated over three different periods. The percentage of inhibition of fungal adhesion to the acrylic strip was calculated using the following formula:

(3) % Inhibition=1−OD490 nm tested OD490 nm control×100%.

### 2.6. XTT Reduction Assay

*C. albicans* viability was determined using a colorimetric XTT reduction assay that measures the activity of mitochondrial dehydrogenase. The XTT solution was prepared by dissolving XTT 0.5 mg/mL in PBS and filter‐sterilizing it through a 0.22‐μm pore filter. XTT solution was mixed with freshly prepared menadione solution (10 mM, prepared in acetone) at a final concentration of 1 µM. XTT–menadione solution was added into each well and incubated in the dark for 3 h at 37°C. After incubation, the supernatant (100 μL) was transferred to new microtiter plates and the optical density was measured at 490 nm with a microtiter plate reader (Bio‐Rad Laboratories Inc., Hercules, CA, USA).

### 2.7. Inhibitory Effect Against GTF

The effect of EO and PE on GTF was performed as previously described [17]. GTF was induced in SDB containing fetal bovine serum (FBS) (10% v/v) (Gibco‐BRL, Grand Island, NY, USA). Briefly, 100 μL of *C. albicans* suspension (∼1 × 10^7^ cells/mL) was inoculated in 2 mL of SDB containing FBS without or with EO or PE. *C. albicans* ATCC10231 was treated with EO or PE at MIC, 2×MIC, and 4×MIC, whereas the clinical isolate was exposed to different concentrations at 0.25×MIC and 0.5×MIC. Samples were incubated at 37 °C for 3 h, and then, total yeast cells and GTF were determined by counting 300 cells using a hemocytometer chamber under a microscope (ZEISS Axio Observer Z1). GTF counting criteria included (a) yeast cells with a germ tube without any constriction at the junction between the mother cell and tail and (b) yeast cells with the tail equal to or longer than the mother cell diameter. Clump cells with germ tubes and pseudohyphae were excluded [18]. SDB with FBS without treatment or treated with CHX was designated as the control or the positive control, respectively. Each test was performed in triplicate, and the experiments were repeated over three different periods of time. The percentage of germination reduction was calculated using the following formula:

(4) % Reduction=1− No. GTF tested No. GTF control×100%.

### 2.8. Statistical Analysis

The results were expressed as means and standard deviations (mean ± SD). Differences in *Candida* cell viability, *C. albicans* adhesion to denture acrylic, and GTF among the groups were analyzed using one‐way analysis of variance (ANOVA). Statistical significance was defined as *p* < 0.05.

## 3. Results

### 3.1. Inhibition and Eradication of Biofilm Formation

The effect of EO and PE on the inhibition of biofilm formation and eradication of established biofilm is presented in Table 1. Among the five tested samples, cinnamon bark oil had a significant effect, with the lowest MBIC_90_ (0.156 mg/mL) and MBEC_90_ (0.313 mg/mL) against both *C. albicans* strains (*p* < 0.05), followed in decreasing order of effectiveness by lemongrass oil, clove bud oil, *A. galanga* extract, and *P. betle* extract. Furthermore, all tested samples consistently inhibited biofilm formation and eradicated established biofilms in a concentration‐dependent manner (Figure 1). All samples achieved almost complete inhibition and eradication of the biofilm (> 99%) against both *C. albicans* strains. The only exception was *P. betle* extract at 5 mg/mL, which exhibited lower effectiveness, less than 90% inhibition and eradication of fungal biofilms, against the clinical strain (*p* < 0.05).

**Table 1:** Effect of essential oils and plant extracts on inhibition against biofilm formation and eradication of established biofilm of reference and clinical strains of *Candida albicans.*

Essential oils/plant extracts	*C. albicans* ATCC10231		*C. albicans* R01	
	MBIC_90_ (mg/mL)	MBEC_90_ (mg/mL)	MBIC_90_ (mg/mL)	MBEC_90_ (mg/mL)
Cinnamon bark oil	0.156	0.313	0.156	0.313
Lemongrass oil	0.313	0.625	0.313	0.625
Clove bud oil	1.250	1.250	1.250	1.250
*P. betle* extract	2.500	5.000	5.000	> 5.000
*A. galanga* extract	2.500	5.000	2.500	5.000
Chlorhexidine (CHX)	0.031	0.031	0.031	0.031

*Note:* MBIC_90_, the minimum biofilm inhibitory concentration at 90%; MBEC_90_, the minimum biofilm eradication concentration at 90%.

Figure 1Effects of essential oils and plant extracts on *Candida albicans* biofilm formation and eradication. (a, c) *C. albicans* ATCC1023 and (b, d) clinical strain R01. (a, b) Biofilm formation inhibition; (c, d) established biofilm eradication. The results were expressed as means and standard deviations of three independent experiments.

**Figure:**
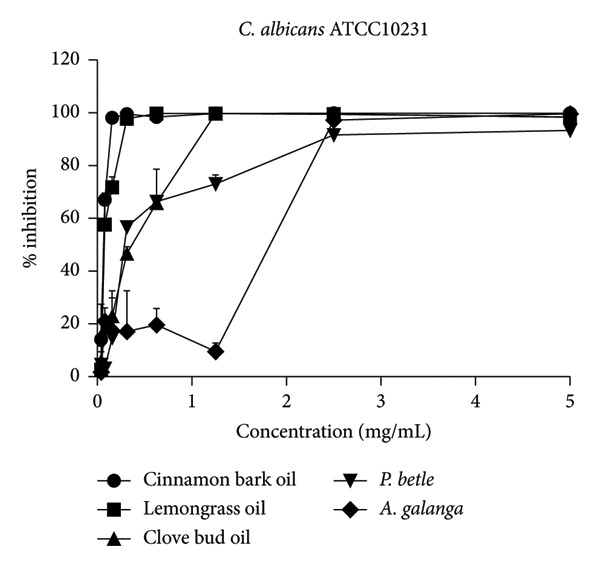
(a)

**Figure:**
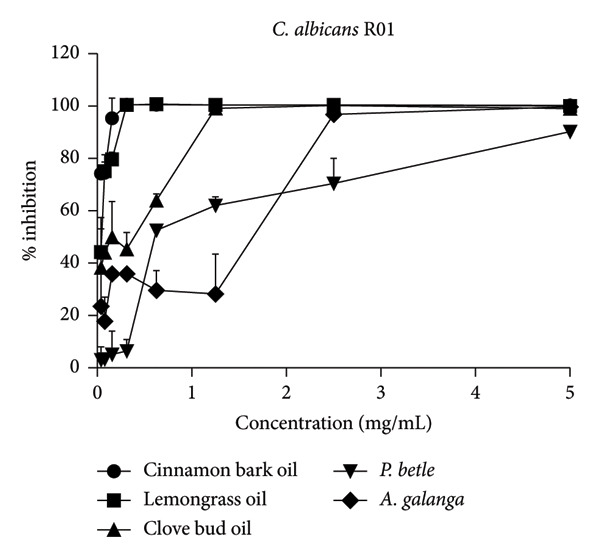
(b)

**Figure:**
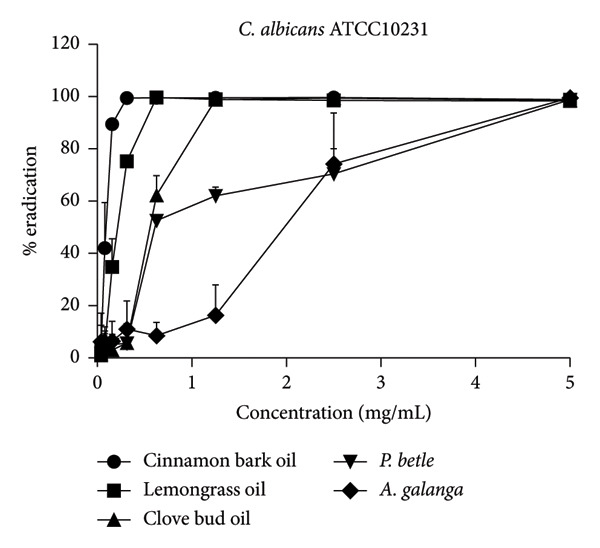
(c)

**Figure:**
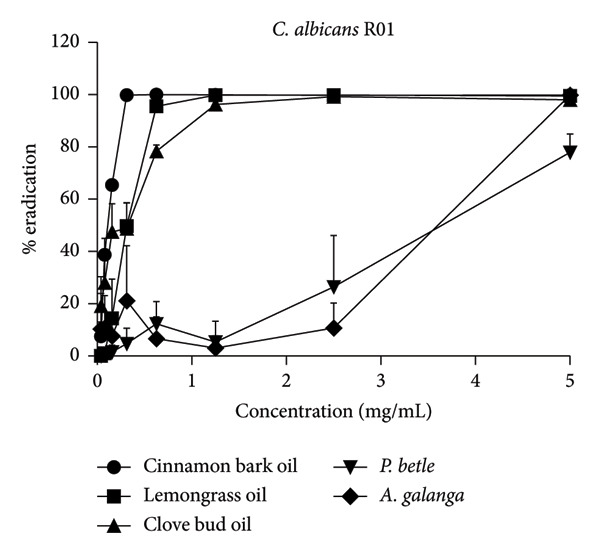
(d)

### 3.2. Effect on Yeast Cell Adhesion

Both EO and PE inhibited yeast cell adhesion to varying degrees (Table 2). Most EO and PE at 4×MIC significantly reduced *C. albicans* adhesion to acrylic by 70%–90% for both reference and clinical strains. Exceptions were clove bud oil and *P. betle* extract, which showed weaker inhibition (∼40%–50%). Interestingly, *A. galangal* extract at 2×MIC was sufficient to reduce cell adhesion by approximately 80% for both strains. In addition, all treatments demonstrated dose‐dependent inhibitory effects on adhesion.

**Table 2:** Inhibition effect of essential oils and plant extracts on *Candida albicans* ATCC10231 and *C. albicans* R01 adhesion to denture acrylic.

Essential oils/plant extracts	Concentrations (mg/mL)	% Inhibition of *C. albicans* adhesion to denture acrylic	
		ATCC10231	R01
Cinnamon bark oil	0.625	ND	71.52 ± 9.58^AB^
	0.313	77.33 ± 4.16^A^	50.16 ± 6.32^C^
	0.156 (MIC)^b^	47.00 ± 1.73^B^	31.07 ± 3.36^C^
	0.078 (MIC)^a^	17.00 ± 6.08^C^	ND
Lemongrass oil	1.250	ND	72.49 ± 2.97^AB^
	0.625	78.62 ± 8.43^A^	57.28 ± 11.77^BC^
	0.313 (MIC)^b^	61.63 ± 5.65^AB^	34.30 ± 2.44^C^
	0.156 (MIC)^a^	40.90 ± 13.14^BC^	ND
Clove bud oil	2.500	ND	39.48 ± 7.02^C^
	1.250	74.29 ± 5.38^A^	36.57 ± 9.02^C^
	0.625 (MIC)^b^	68.64 ± 4.24^AB^	6.47 ± 5.85^D^
	0.313 (MIC)^a^	29.38 ± 14.93^BC^	ND
*P. betle* extract	5.000	ND	37.64 ± 7.15^C^
	2.500	ND	12.70 ± 2.58^CD^
	1.250 (MIC)^b^	53.67 ± 6.25^AB^	2.49 ± 4.32^D^
	0.625	18.93 ± 7.78^C^	ND
	0.313 (MIC)^a^	0.56 ± 0.98^D^	ND
*A. galanga* extract	10.00	ND	86.41 ± 6.80^A^
	5.000	85.31 ± 4.87^A^	88.03 ± 2.44^A^
	2.500 (MIC)^b^	80.75 ± 7.62^A^	90.29 ± 5.14^A^
	1.250 (MIC)^a^	41.81 ± 13.45^B^	ND
Chlorhexidine (CHX)	0.128	ND	93.85 ± 2.97^A^
	0.064	83.83 ± 0.15^A^	86.73 ± 10.38^A^
	0.032 (MIC)^b^	56.54 ± 8.69^AB^	69.90 ± 7.95^BC^
	0.016 (MIC)^a^	34.26 ± 11.18^BC^	ND

*Note:* Means denoted by the different capital letters (A–D) in each column are significantly different at *p* < 0.05.

Abbreviation: ND, not determined.

^a^MIC value of the tested agent on *C. albicans* ATCC10231 (reference strain).

^b^MIC value of the tested agent on *C. albicans R01* (clinical strain).

### 3.3. Inhibitory Effect Against GTF

The inhibitory effect of EO and PE against GTF of *C. albicans* is presented in Table 3. Most EO and PE achieved greater than 90% inhibition of *C. albicans* GTF at concentrations of 2×MIC for the reference strain and MIC for the clinical strain, respectively. The exception was *P. betle* extract, which only showed inhibition against the clinical strain. Furthermore, at 0.5×MIC, *A. galanga* extract, cinnamon bark oil, and lemongrass oil inhibited GTF of the clinical *C. albicans* strain by 70%–90%. In comparison, *P. betle* and clove bud oil inhibited the fungal GTF by 50%–58% (Figure 2).

**Table 3:** Reduction in germination of *Candida albicans* ATCC10231 and *C. albicans* R01 after exposure to various concentrations of essential oils and plant extracts.

Essential oils/plant extracts	Concentration (mg/mL)	% Reduction in germination of *C. albicans*	
		ATCC10231	R01
Cinnamon bark oil	0.313	98.82 ± 1.02^A^	ND
	0.156 (MIC)^b^	97.30 ± 2.13^A^	99.54 ± 0.80^A^
	0.078 (MIC)^a^	56.73 ± 7.09^C^	76.62 ± 10.37^B^
	0.039	ND	21.56 ± 11.95^D^
Lemongrass oil	0.625	99.18 ± 0.73^A^	ND
	0.313 (MIC)^b^	96.94 ± 1.24^A^	99.54 ± 0.80^A^
	0.156 (MIC)^a^	75.43 ± 9.01^B^	92.97 ± 6.67^AB^
	0.078	ND	48.28 ± 14.09^C^
Clove bud oil	1.250	99.76 ± 0.20^A^	ND
	0.625 (MIC)^b^	96.24 ± 2.85^A^	98.96 ± 1.80^A^
	0.313 (MIC)^a^	46.15 ± 2.35^C^	58.65 ± 10.89^BC^
	0.152	ND	9.24 ± 3.88^D^
*P. betle* extract	2.500 (MIC)^b^	ND	90.56 ± 1.31^A^
	1.250	31.92 ± 7.95^D^	51.28 ± 4.65^C^
	0.625	15.23 ± 4.09^E^	17.99 ± 0.87^D^
	0.313 (MIC)^a^	5.82 ± 4.85^E^	ND
*A. galanga* extract	5.000	99.41 ± 1.02^A^	ND
	2.500 (MIC)^b^	97.53 ± 2.15^A^	100.00 ± 0.00^A^
	1.250 (MIC)^a^	94.12 ± 1.13^A^	72.13 ± 5.96^B^
	0.625	ND	45.40 ± 14.86^C^
Chlorhexidine (CHX)	0.064	98.82 ± 1.02^A^	ND
	0.032 (MIC)^b^	97.30 ± 1.24^A^	99.65 ± 0.60^A^
	0.016 (MIC)^a^	74.84 ± 4.76^B^	93.20 ± 7.45^AB^
	0.008	ND	4.52 ± 2.45^D^

*Note:* Means denoted by the different capital letters (A–E) in each column are significantly different at *p* < 0.05.

Abbreviation: ND, not determined.

^a^MIC value of the tested agent on *C. albicans* ATCC10231 (reference strain).

^b^MIC value of the tested agent on *C. albicans* R01 (clinical strain).

Figure 2Effect of natural extracts on germ tube formation of *Candida albicans* R01 (clinical strain). In culture medium: (a) at 0 min; (b) after 3‐h incubation in culture medium; and after 3‐h incubation at 0.5×MIC with (c) cinnamon bark oil, (d) lemongrass oil, (e) clove bud oil, (f) *P. betle* extract, (g) *A. galanga* extract, and (h) chlorhexidine (200× magnification; bar = 20 μm).

**Figure:**
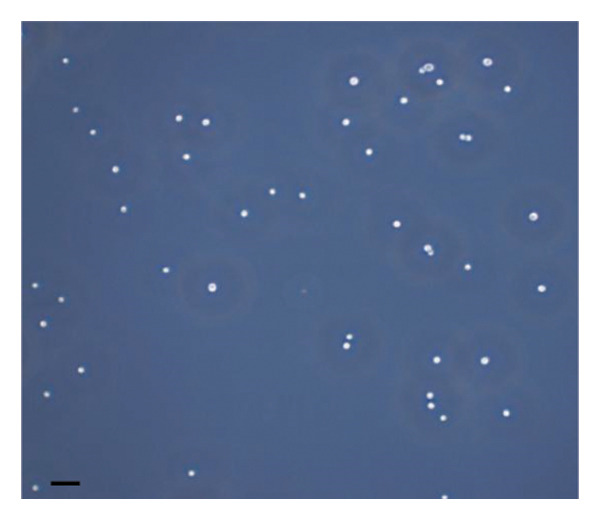
(a)

**Figure:**
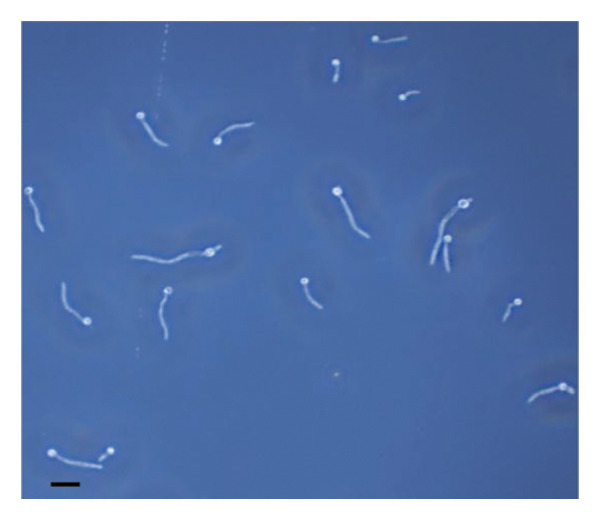
(b)

**Figure:**
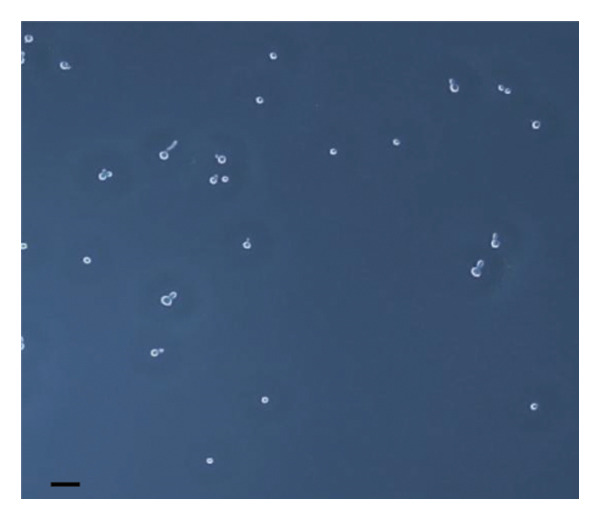
(c)

**Figure:**
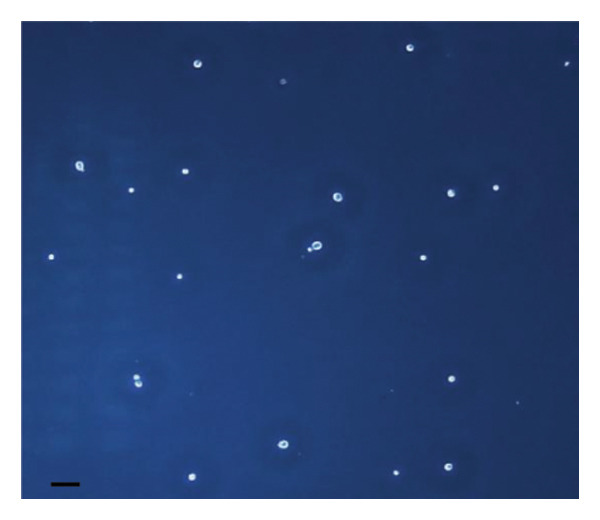
(d)

**Figure:**
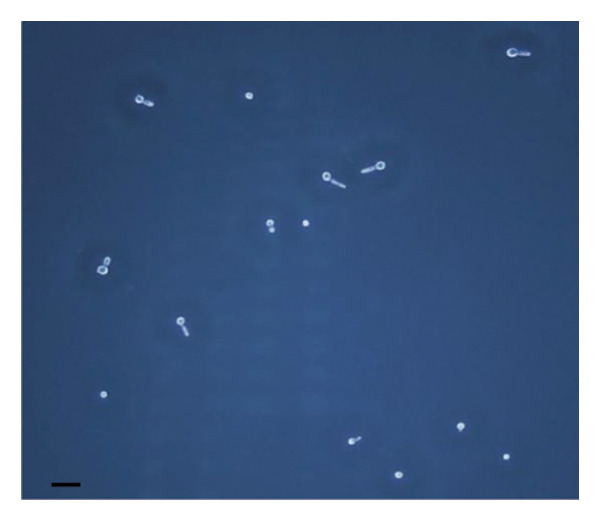
(e)

**Figure:**
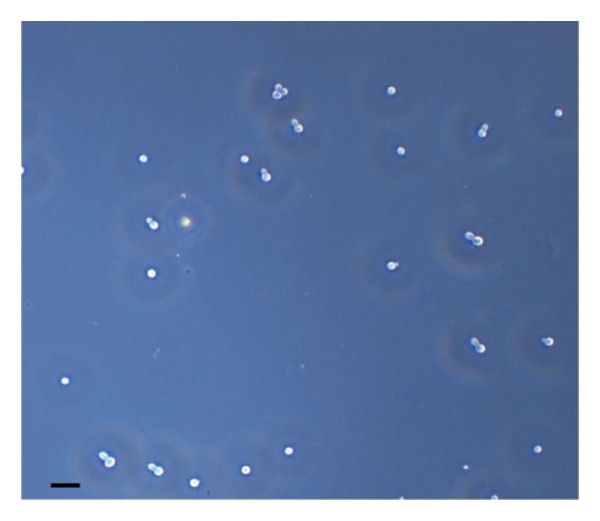
(f)

**Figure:**
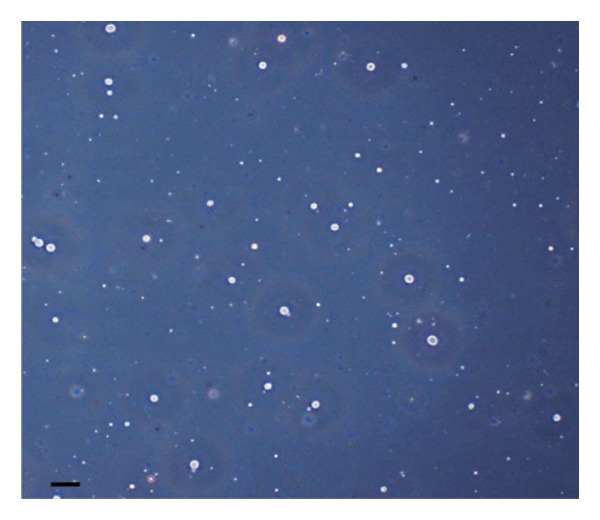
(g)

**Figure:**
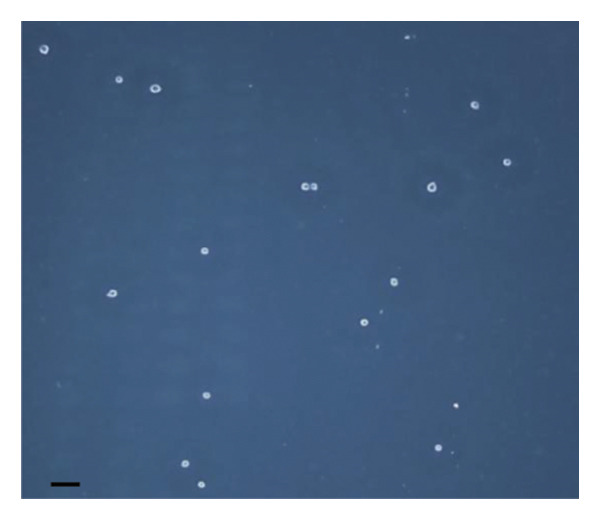
(h)

## 4. Discussion

The development of *C. albicans* biofilm involves three critical phases including adhesion, initial colonization, and maturation [19]. Consequently, *C. albicans* biofilms exhibit distinct characteristics from their planktonic counterparts, including resistance to antifungal drugs [9]. Therefore, effective natural anti–*Candida* products should inhibit fungal adhesion, yeast–hyphae transition, and biofilm formation. Three EOs (cinnamon bark, lemongrass, and clove bud) and two PEs (*P. betle* and *A. galanga*) were investigated for their anti–*Candida* properties including inhibition and eradication of fungal biofilm, reduction in fungal adhesion, and inhibition of GTF, in this study.

The present study showed that all tested agents exhibited potent effects in inhibiting biofilm formation and eradicating established biofilm of both *C. albicans* reference and clinical strains. These findings agreed with the previous study reporting that cinnamon bark oil, lemongrass oil, and clove bud oil were effective against biofilm formation with MBIC_90_ and/or MBEC_90_ values against *C. albicans* in a range of 2.500–5.000 μL/mL, 0.8–1.6 mg/mL, and 5.00–10.00 μL/mL, respectively [12, 18]. The findings were also supported by the previous investigations that major compounds such as cinnamaldehyde (cinnamon bark oil), citral (lemongrass oil), and eugenol (clove bud oil) inhibited fungal biofilm formation as well as eradicated established fungal biofilm [20, 21]. However, much lower MBIC_90_ (0.156 mg/mL, 0.313 mg/mL, and 1.230 mg/mL) and MBEC_90_ (0.313 mg/mL, 0.625 mg/mL, and 1.250 mg/mL) values of these three EOs were found in this present study, compared to the previous studies. In addition to the EO tested, *P. betle* extract expressed anti–*Candida* biofilm properties. The MBIC_90_ (2.50 mg/mL) and MBEC_90_ (5.0–> 5.0 mg/mL) of this PE were much lower than those reported previously. Kawsud et al. observed that the MBIC_90_ and MBEC_90_ of *P. betle* ethanolic extract against *C. albicans* ATCC 90028 were 3.13 ± 0.15 and 12.50 ± 0.69 mg/mL, respectively [11]. Phumat et al. reported that the anti–*Candida* biofilm property of hydroxychavicol compound extracted from *P. betle* against *C. albicans* DMST 8684 was 400–1000 μg/mL [22]. This study was also the first report on the inhibitory effect of *A. galanga* extract against *C. albicans* biofilm formation and eradication against established fungal biofilm. The present results demonstrate that all tested EO and PE contain effective anti–*Candida* biofilm activities, especially cinnamon bark oil, lemongrass oil, and *A. galangal* extract. These findings are supported by our previous study (Table S1), which identified bioactive compounds present in EO and PE using gas chromatography–mass spectrometry (GC–MS). The major constituents were geranial and neral in lemongrass oil, eugenol in clove bud oil, cinnamaldehyde in cinnamon bark oil, 4‐allyl‐1,2‐diacetoxybenzene and hydroxychavicol in *P. betle* extract, and 1′‐acetoxychavicol acetate in *A. galanga* extract [14]. Interestingly, the MBIC_90_ values of cinnamon bark oil, lemongrass oil, and *A. galangal* extract were identical to their MIC values. Therefore, the concentration at MIC of these natural‐derived products has interfered with the formation of fungal biofilm. These results imply that the tested EO and PE may affect the adhesion of yeast cells to acrylic and true hyphae formation [23]. Moreover, both the inhibition of biofilm formation and the eradication of established fungal biofilm of all tested EO and PE were dose‐dependent.

*Candida* adhesion to denture acrylic is a crucial step to initiate and propagate fungal biofilm causing DS [24]. The current study found that the adhesion of *C. albicans* to denture acrylic was reduced after exposure to EO and PE at the tested concentration. These findings were supported by previous studies demonstrating that the main active compounds in EO inhibited *C. albicans* adhesion [25–27]. Surprisingly, > 80% reduction in the adhesion of cells to denture acrylic was observed after exposure to *A. galanga* extract at concentrations of 2.500 mg/mL, similar to those of commercial CHX at a concentration of 0.064 mg/mL. This present study is the first report that *A. galanga* extract may be used as effectively as denture cleansers available commercially. However, *P. betle* extract showed little effect on the adhesion of yeast cells to denture acrylic for both *C. albicans* strains even after exposure to 4×MIC (5.000 mg/mL). However, it has been reported that the ethanolic leaf extract of *P. betle* at a concentration of 8 mg/mL has a similar effect on the adhesion of *C. albicans* ATCC 90028 to denture acrylic surfaces as commercial denture cleanser [28]. This may be due to the lipophilic properties of EO, and *A. galanga* had an effect on the surface of the *C. albicans* membrane, potentially altering its properties and affecting the yeast’s adhesion capabilities [29–33]. These results found that the inhibition of fungal adherence to acrylic in all tested samples was consistent with the prevention of biofilm formation and elimination of mature biofilm in all tested samples.

The morphological transition of *C. albicans* plays a crucial role in the initial colonization phase of biofilm formation and mammalian cell penetration. The hyphae cell can attach to the denture surface or penetrate the oral mucosa, damaging epithelial cells and causing DS symptoms [34, 35]. The present investigation and the concentration of tested oil and PE at 0.5×MIC were also tested to ensure that the inhibition of GTF is not due solely to growth inhibition. The results showed that all EOs can inhibit more than 90% of GTF on both *C. albicans* strains. This finding was supported by the previous investigation, which showed that the major compounds in all EO exhibited strong inhibitory effects on *C. albicans* germination [21, 26]. Moreover, the *A. galanga* extract has the potential to completely inhibit the transition from yeast to the hyphae form of both *C. albicans* strains. Similar results had been reported that the ethanolic extract of *A. galanga* at a concentration of 1 mg/mL was able to inhibit the germ tube of *C. albicans* [36]. In contrast, *P. betle* extract exhibited little effect on GTF in the reference strain even after exposure to 4×MIC. From the present study, it is evident that lemongrass oil, cinnamon bark oil, and *A. galanga* extract at low concentrations below the MIC (0.5×MIC) were able to inhibit the germination on the clinical isolate of *C. albicans* (Figure 2). This effect may be due to interaction with cellular lipid components and cell membrane, leading to changes and loss of the structural and enzymatic constituents of fungal cells that are required in GTF for its growth [31–33]. Therefore, these findings indicate that all tested agents, especially cinnamon bark oil, lemongrass oil, and *A. galanga* extract, could effectively inhibit biofilm formation through decreasing adhesion and yeast–hyphae transition of *C. albicans* cells.

Regarding the cytotoxicity assessment, previous studies have already demonstrated the low toxicity of cinnamon bark oil, lemongrass oil, and *A. galanga* extract to human cells, indicating their safety for use. Cinnamon bark oil at 0.5% demonstrated no toxicity to human gingival fibroblast cells for up to 24 h [37]. Another study reported that lemongrass oil at a concentration of 0.625 mg/mL was both effective against *C. albicans* biofilms and nontoxic to human cells [38]. The ethanolic extract of galangal (1000 μg/mL) demonstrated no cytotoxicity toward human keratinocytes and fibroblasts, suggesting its safety for topical applications [39]. Moreover, our previous work reported that the mouthwash powders containing *A. galanga* extract and lemongrass oil loaded into a self‐nanoemulsifying drug delivery system (SNEDDS) showed no toxicity to normal human fibroblast cells [40].

## 5. Conclusion

This study concluded that all tested agents, especially cinnamon bark oil, lemongrass oil, and *A. galanga* extract, exhibited strong inhibitory effects on the cell adhesion, morphological transition, and biofilm formation of both *C. albicans* reference and clinical strains. Therefore, these EO and PE represent potential natural‐derived antifungal agents that could be used in oral hygiene products for DS management. Nevertheless, further investigations including cytotoxicity on buccal cells and the effect of these EO and PE on the physical properties of denture acrylic resin should be explored before the development of plant‐based dental products to promote oral hygiene.

## Conflicts of Interest

The authors declare no conflicts of interest.

## Author Contributions

Premnapa Sisopa conceptualized the study, curated the data, involved in formal analysis, acquired funding, investigated the data, designed the methodology, provided resources, validated the data, visualized the data, and wrote the original draft. Supaporn Lamlertthon conceptualized the study, acquired funding, designed the methodology, provided resources, and validated, wrote, reviewed, and edited the manuscript. Ruchadaporn Kaomongkolgit conceptualized the study, acquired funding, designed the methodology, provided resources, and validated, wrote, reviewed, and edited the manuscript. Pratthana Chomchalao conceptualized the study, designed the methodology, provided resources, visualized the data, and wrote, reviewed, and edited the manuscript. Waree Tiyaboonchai conceptualized the study, acquired funding, designed the methodology, administered the project, supervised the data, validated the data, and wrote, reviewed, and edited the manuscript.

## Funding

This study was funded by Naresuan University (NU), Thailand Science Research and Innovation (TSRI), National Science Research and Innovation Fund (NSRF) (Fundamental Fund: Grant No. R2566B016), and Thailand’s Ministry of Science and Technology (MOST) under the Thai Government Science and Technology Scholarship.

## Supporting information


**Supporting Information 1** Supporting Information. Table S1: The major compounds of essential oils and plant extracts determined by GC–MS.

## Data Availability

The data supporting this study’s findings are available on request from the corresponding author.
