# Supplementary material for: Multitarget Anti‐Candida Activity of Thai Plant Extracts and Essential Oils: Inhibiting Biofilm Formation, Denture Adhesion, and Germ Tube Formation
Source: Scientifica (Cairo). 2025 Dec 10;2025:1766872. doi: 10.1155/sci5/1766872 (PMC12782332; doi:10.1155/sci5/1766872)
Supplement: Supplementary file 1 — Supporting Information 1 Supporting Information. Table S1: The major compounds of essential oils and plant extracts determined by GC–MS. [file SCI5-2025-1766872-s001.docx]

**Supplementary Information**

**Table S1** The major compounds of essential oils and plant extracts determined by GC-MS

| **plant extracts/ Essential oils** | **Main compounds** | **Relative area (%)** |
| --- | --- | --- |
| Cinnamon bark oil | Cinnamaldehyde | 80.6 |
|  |  |  |
| Clove bud oil | Eugenol | 85.5 |
|  |  |  |
| Lemongrass oil | Alpha-citral (Geranial) | 42.7 |
|  | Beta-citral (Neral) | 22.2 |
|  |  |  |
| *P. betle* extract | 4-allyl-1,2-diacetoxybenzene | 29.5 |
|  | Hydroxychavicol | 24.8 |
|  | Eugenol | 24.7 |
|  | Eugenol acetate | 21.0 |
|  |  |  |
| *A. galanga* extract | 1’-acetoxychavical acetate | 78.0 |

Relative area, compound percentages were obtained electronically from the GC-MS percent area data.
